# Case series on resection of symptomatic bifid ribs: A successful and safe treatment option

**DOI:** 10.1016/j.ijscr.2024.110025

**Published:** 2024-07-10

**Authors:** Jelle A. Nieuwstraten, Pieter J. van Huijstee

**Affiliations:** a Department of Surgery, Leiden University Medical Centre, Leiden, the Netherlands; b Department of Surgery, Haga Teaching Hospital, The Hague, the Netherlands

**Keywords:** Bifid rib, Forked rib, Surgery, Symptomatic

## Abstract

**Introduction:**

Bifid ribs are a type of, generally asymptomatic, congenital chest wall abnormality. However, patients sometimes complain about chronic chest pains, deformities and respiratory difficulty. There is limited literature regarding treatment of symptomatic bifid ribs. We present the results of two cases of symptomatic bifid ribs causing intercostal nerve impingement who underwent surgery.

**Presentation of case:**

Two patients aged 22 and 33 presented to the outpatient clinic with chronic chest pains. After physical examination and 3D CT-scans they were diagnosed with intercostal nerve impingement caused by a bifid rib. Both patients underwent surgery to resect the upper arch of the bifid rib. Upon follow-up nine and seven months post-operatively patients reported complaints had been completely resolved and follow-up was ended.

**Discussion:**

Although bifid ribs are generally asymptomatic they can sometimes cause intercostal nerve impingement. These cases demonstrate that these symptoms might only start after experiencing minor trauma or a growth spurt. Intercostal nerve blockades might me a useful tool in diagnostic work-up to confirm the diagnosis.

**Conclusion:**

Resection of symptomatic bifid ribs is a safe and viable treatment option.

## Highlights

• Bifid ribs are generally asymptomatic but can cause intercostal nerve impingement
• In cases of intercostal nerve impingement surgery is a successful treatment option
• Intercostal nerve blockades can be used during diagnostic work-up

## 1 Introduction

Bifid ribs, sometimes called forked ribs, make up 28 % of costal abnormalities. They mostly occur unilaterally and right-sided. Bifurcation is most often seen in the third and fourth rib [1,2]. The intercostal space above the bifid rib is often narrowed whereas the lower intercostal space is widened [3]. Bifid ribs are generally asymptomatic and therefore only diagnosed as incidental findings on radiological examination or cadaveric dissection [1,3]. However, various case reports describe patients with symptomatic bifid ribs experiencing chest pains, chest wall deformity or respiratory difficulty [1,2,4]. Literature also describes patients becoming symptomatic after being involved in a minor accident [3]. There is limited literature regarding treatment strategies for symptomatic bifid ribs. To the best of our knowledge there is one other case report, describing a 14-year-old girl with Noonan syndrome who underwent thoracoscopic resection of a symptomatic bifid rib and remained asymptomatic on follow-up [5]. We present the results of two patients who underwent surgery in a local community hospital for symptomatic bifid ribs.

## 2 Presentation of case

**Fig. 1:**
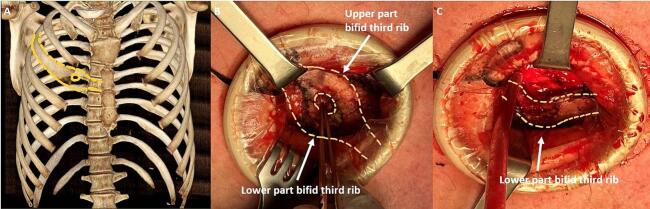
Images of patient A showing 3D reconstruction of CT-scan of thorax with bifid third rib on the right (A), identification of bifid rib intra-operatively (B) and situation after resection of upper part of bifid rib (C).

This work has been conducted in accordance with the PROCESS criteria [6]. The local ethics review board does not require ethical approval or clearance for case reports and series. Patient A is a 22-year-old male with a prior history of scoliosis. He presented himself to our outpatient clinic due to chronic chest pain on the right ventral side. Complaints first started during his growth spurt at age 14 and worsened over time. He had previously visited an orthopaedic surgeon who determined the complaints were not caused by scoliosis. Conventional X-rays did not find any explanation for the complaints. There was no trauma in his history. Upon physical examination there were no visible or palpable deformities on his chest. However, compression of the third rib on the right side of the sternum caused pain. Paracetamol and NSAID's did not alleviate symptoms. Computed tomography (CT) with three-dimensional (3D) reconstruction of the thorax revealed a bifid rib of the third rib on the right side, correlating with the painful location during physical examination (Fig. 1A). The upper arch of the bifid rib caused the second intercostal space to be narrowed. The patient was therefore diagnosed with impingement of the second intercostal nerve. After shared decision-making he chose to undergo surgery to remove the upper part of the bifid rib to release the second intercostal nerve. An incision was made over the affected rib directly parasternal and the bifid rib was identified (Fig. 1B). The pectoralis major was incised longitudinally using diathermy. The upper arch of the bifid rib and the lower border of the second rib were dissected using diathermy, leaving the pleural cavity intact, after which the upper arch was transected medially using an osteotome. The upper part was then dissected laterally towards the connection with the lower arch, transected there and removed. This resulted in a widened second intercostal space and release of the second intercostal nerve. The lower arch was left in place (Fig. 1C). The intercostal neurovascular bundle was protected using blunt instruments during dissection. The intercostal muscle, pectoralis major, subcutaneous layer and skin were closed using absorbable sutures. Post-operative pain was managed using paracetamol and NSAID's. The patient was discharged the same day and an appointment was scheduled for 6 weeks and 3 months post-operatively. He reported to be asymptomatic on these visits. Nine months postoperatively a last phone call appointment was conducted and the patient reported complaints had not recurred and follow-up was ended.

**Fig. 2:**
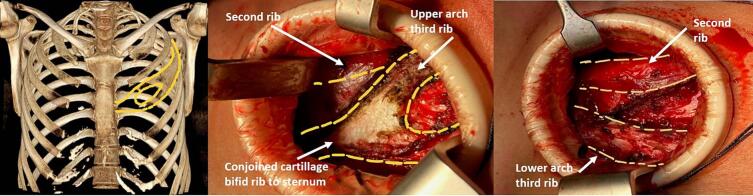
Images of patient B showing 3D reconstruction of CT-scan of thorax with bifid third rib on the left (A), identification of bifid rib intra-operatively (B) and situation after resection of upper arch of bifid rib (C).

Patient B is a 33-year-old female without prior medical history. She was referred to our outpatient clinic because of chronic thoracic pain which started after being involved in a minor motor vehicle accident (without injury) two years earlier. She described experiencing pain on the left side of her sternum, radiating to her left arm during deep breathing and movement. Paracetamol and NSAID's did not alleviate symptoms. A bulge was visible and palpable directly left of the sternum on the level of the third rib. Palpation of the bulge elicited the same pain she described. Conventional thoracic X-rays did not find any explanation for the complaints. A CT-scan with 3D-reconstruction of the thorax revealed a bifid rib of the third rib on the left, correlating with the painful site upon physical examination, consisting of an upper and lower arch with a narrowed second intercostal space (Fig. 2A). She was diagnosed with impingement of the second intercostal nerve caused by the upper arch. An intercostal nerve block was performed to determine if complaints were indeed caused by intercostal nerve impingement. Pain was alleviated for a short period of time but eventually returned. After shared decision-making it was decided to remove the upper arch. The same surgical approached was used as in patient A. An incision was made directly parasternal over the bulge and the upper arch of the bifid rib was identified (Fig. 2B). The upper arch was removed after which the second intercostal space was widened and the intercostal nerve released (Fig. 2C). Post-operative pain was managed using paracetamol and NSAID's. The patient was discharged the same day and an appointment was scheduled for 6 weeks and 3 months post-operatively. She reported to be asymptomatic on these visits. Seven months postoperatively a last phone call appointment was conducted and the patient reported complaints had not recurred and follow-up was ended.

## 3 Discussion

These cases demonstrate that patients with bifid ribs can experience chronic chest pains due to intercostal nerve impingement. They were asymptomatic up until their growth spurt started (patient A) or were involved in a minor accident (patient B). This illustrates certain events can provoke bifid ribs becoming symptomatic, consistent with current literature [3]. Intercostal nerve impingement caused by bifid ribs should be suspected if patients describe experiencing chronic pain which they can clearly localize. Upon physical examination the pain can be elicited by compression of the affected rib. A bulge might be clearly palpable or visible. 3D CT-imaging can reveal the bifid rib, correlating with the painful location upon physical examination, after which an intercostal nerve block might be useful pre-operatively to determine if complaints are indeed caused by impingement. Both patients were extensively counselled regarding various other treatment options, including conservative treatment with nerve blockades and oral painkillers. As described, patient B underwent an intercostal nerve block prior to surgery which was unsuccessful in achieving long term relief of complaints. Since complaints in both patients had existed for a long time they both elected to undergo surgery. In these cases patients had a pronounced benefit of surgery and no complications occurred. This is in line with the only other known case report regarding surgical treatment of a bifid rib [5]. In the current article the bifid ribs were resected via a direct open procedure, as opposed to a thoracoscopic resection presented in the paper of Delgado-Miguel et al., demonstrating this is a viable treatment option as well.

## 4 Conclusion

Partial resection of a bifid rib in case of impingement is a safe and effective treatment option with good results concerning pain relief. Since literature is scarce there is no data regarding complications of this surgery. Possible complications could include wound infections and intercostal nerve damage or pneumothorax caused by the procedure.

## Funding

None.

## Consent

Written informed consent was obtained from the patient for publication of this case report and accompanying images. A copy of the written consent is available for review by the Editor-in-Chief of this journal on request.

## CRediT authorship contribution statement

J.A. Nieuwstraten – Writing (Original Draft), Writing (review, editing), Visualization.

P.J. van Huijstee – Investigation, Resources, Writing (review, editing), Visualization, Supervision.

## Conflicts of interest

None.

## Data Availability

Underlying data will be shared on reasonable request to the corresponding author.
